# Characterization of the promoter region of the bovine *SIX1* gene: Roles of MyoD, PAX7, CREB and MyoG

**DOI:** 10.1038/s41598-017-12787-5

**Published:** 2017-10-03

**Authors:** Da-wei Wei, Xue-yao Ma, Song- Zhang, Jie-yun Hong, Lin-sheng Gui, Chu-gang Mei, Hong-fang Guo, Li- Wang, Yue- Ning, Lin-sen Zan

**Affiliations:** 1 College of Animal Science and Technology, Northwest A&F University, Yangling, 712100 Shaanxi, People’s Republic of China; 2 National Beef Cattle Improvement Center, Northwest A&F University, Yangling, 712100 Shaanxi, People’s Republic of China; 3 Modern Cattle Biotechnology and Application of National-Local Engineering Research Center, Yangling, 712100 Shaanxi, People’s Republic of China; 4 Shaanxi Beef Cattle Engineering Research Center, Yangling, 712100 Shaanxi, People’s Republic of China

## Abstract

The *SIX1* gene belongs to the family of six homeodomain transcription factors (TFs), that regulates the extracellular signal-regulated kinase 1/2 (ERK1/2) pathway and mediate skeletal muscle growth and regeneration. Previous studies have demonstrated that *SIX1* is positively correlated with body measurement traits (BMTs). However, the transcriptional regulation of *SIX1* remains unclear. In the present study, we determined that bovine *SIX1* was highly expressed in the *longissimus thoracis*. To elucidate the molecular mechanisms involved in bovine *SIX1* regulation, 2-kb of the 5′ regulatory region were obtained. Sequence analysis identified neither a consensus TATA box nor a CCAAT box in the 5′ flanking region of bovine *SIX1*. However, a CpG island was predicted in the region −235 to +658 relative to the transcriptional start site (TSS). An electrophoretic mobility shift assay (EMSA) and chromatin immunoprecipitation (ChIP) assay in combination with serial deletion constructs of the 5′ flanking region, site-directed mutation and siRNA interference demonstrated that MyoD, PAX7 and CREB binding occur in region −689/−40 and play important roles in bovine *SIX1* transcription. In addition, MyoG drives *SIX1* transcription indirectly via the MEF3 motif. Taken together these interactions suggest a key functional role for *SIX1* in mediating skeletal muscle growth in cattle.

## Introduction

Skeletal muscle development is a complex process regulated by a multitude of genes and sequence-specific transcription factors (TFs)^1^. In terms of gene regulation, the making of muscle in vertebrates is mainly driven by the action of the myogenic regulatory factor (MRF) family, myoblast determining factors (MyoD), Myf5, myogenin (MyoG) and MRF4^2^. The MRFs serve as myogenic determination factors and control the proliferation and differentiation fate of muscle cells derived from myogenic precursor cells^2^. Sequence-specific TFs, such as myocyte-specific enhancer binding factor 2 (MEF2)^3,4^, paired box 7 (PAX7)^5^ and TEA DNA binding domain factor 4 (TEAD4)^6^, are coordinated in part by the action of MRFs and are regulated by transcriptional activation via the binding of MRFs to promoters. However, MEF3, which is also recognized by TFs of the SIX family, is the most abundant, and the MEF3 element is specifically enriched in promoters targeted by MRF^7^.

The SIX family of TFs includes six members designated SIX1 to SIX6^8^. Among them, SIX1 is localized in both the cytoplasm and the nucleus of mesenchymal stem cells during embryogenesis and is involved in controlling the development of multiple tissue types and organs^9–14^. Importantly, the function of *SIX1* is tied to skeletal muscle development. *SIX1*-null mice die at birth due to hypoplasia and abnormal primary myogenesis caused by the reduction and delayed activation of MRF genes in the limb buds^1,14,15^. Furthermore, *SIX1* drives the transformation of slow-twitch towards fast-twitch (glycolytic) fate during myogenesis development^16^. Consistent with these effects, loss of *SIX1* gene function in zebrafish and mice causes abnormal fast-twitch muscle formation^9,10^. Taken together, these data indicate that *SIX1* is critical for skeletal myogenesis and skeletal muscle development.

Despite the clear role of *SIX1* in regulating the formation of muscles and other tissues, there is limited information regarding the transcriptional regulation of bovine *SIX1* during myogenesis. Exquisitely orchestrated gene expression programmes resulting from the concerted interplay of regulatory elements at promoters and enhancers mediate differentiation and development^17^. In this study, we analysed the molecular mechanisms involved in the regulation of the *SIX1* gene via the 5′ regulatory region. In addition, the coding sequence (CDS) of bovine *SIX1* was cloned, and the relative mRNA expression pattern of bovine *SIX1* in the tissue was determined. Our results provide a solid basis for further research on the regulatory roles of *SIX1* in mediating beef skeletal muscle development.

## Results

### Detection of *SIX1* expression in bovine tissues and organs

**Figure 1:**
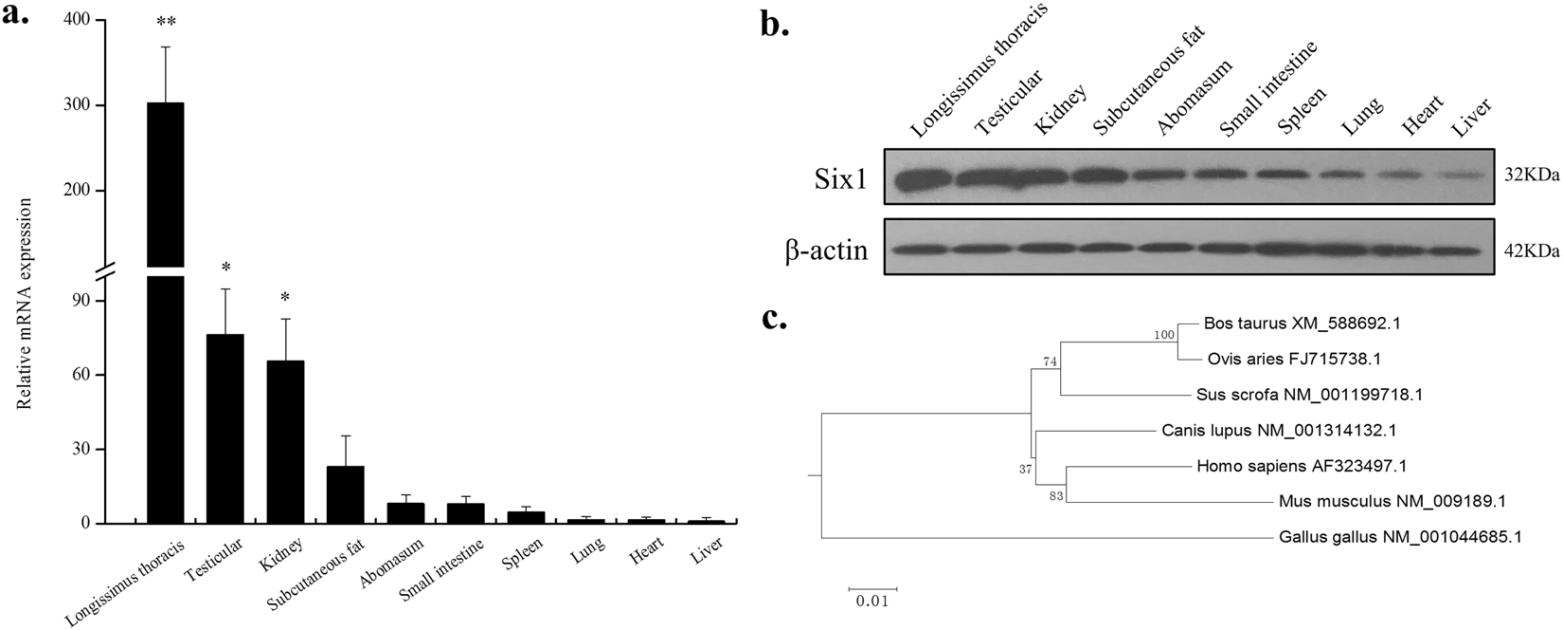
**(a**) Expression pattern analysis of bovine *SIX1* in tissues and organs. *SIX1* mRNA expression was normalized against that of the housekeeping gene *β-actin* and expressed relative to gene expression in the liver. The value of each column represents the mean ± standard deviation based on three independent experiments. Unpaired Student’s t-test was used to detect significant differences. “*”*P* < 0.05 and “**”*P* < 0.01. (**b**) Bovine SIX1 expression pattern at different tissues and organs. (**c**) Phylogenetic tree analysis of *SIX1*. We calculated 8000 bootstrap replicates to bootstrap confidence values.

To detect the role of the bovine *SIX1* gene products in various tissues, cDNA from 10 bovine tissues and organs, including: liver, heart, spleen, lung, kidney, abomasum, small intestine, subcutaneous fat, testicular and *longissimus thoracis* muscle were performed by qPCR (Fig. 1a). The results showed that *SIX1* was predominantly expressed in the *longissimus thoracis* muscle. Moderate *SIX1* expression levels were observed in the testicular tissue and kidney. *SIX1* expression levels were observed slightly in subcutaneous fat, abomasums, small intestine, spleen, lung, heart and liver. The *SIX1* expression level trend remained stable at both the mRNA and protein levels (Fig. 1b and Supplementary Figure S1).

### Molecular cloning and sequence analysis

**Figure 2:**
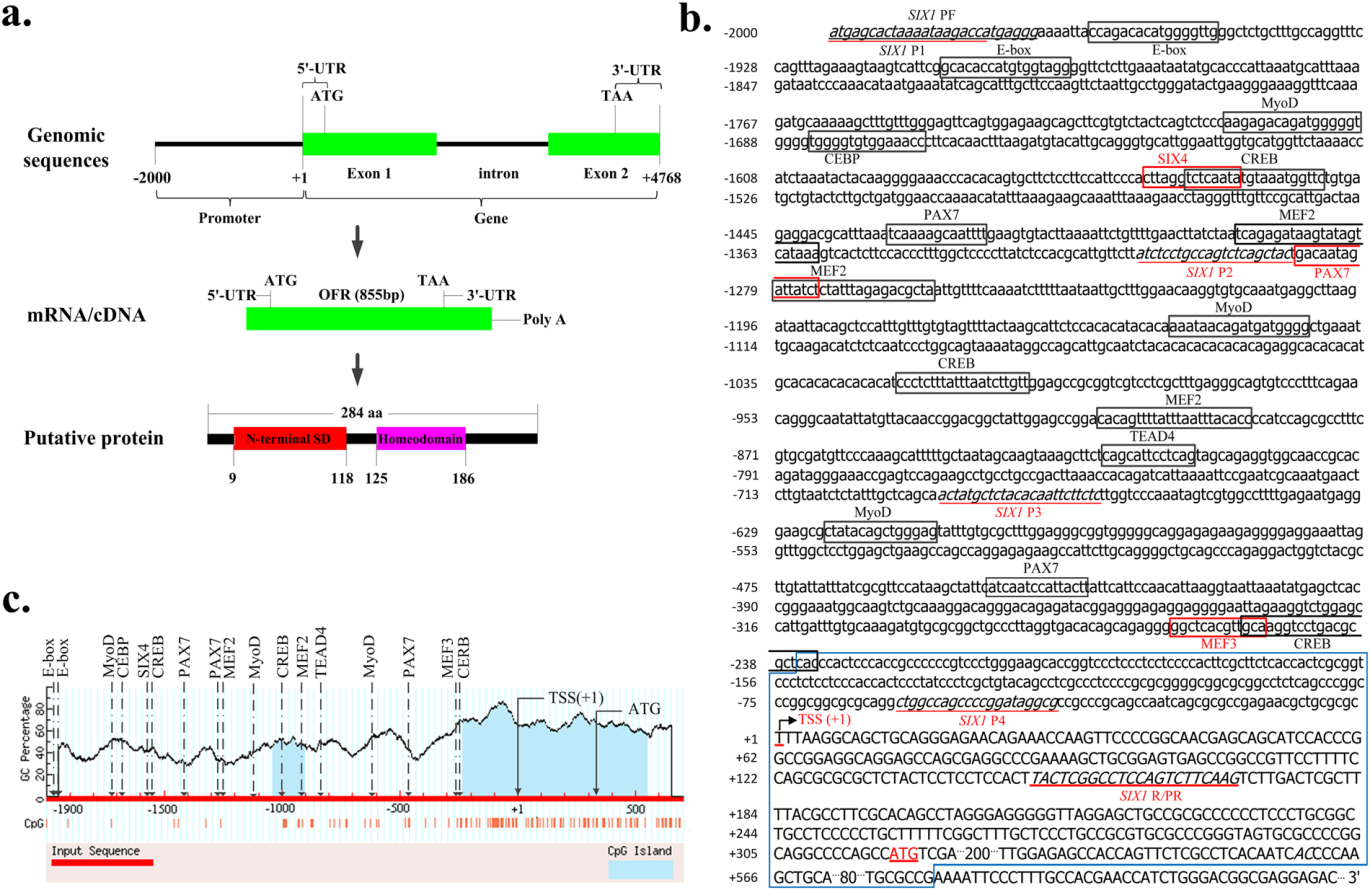
Structural characteristics of the bovine *SIX1* gene. (**a**) The detailed genomic, mRNA and protein components containing the 5′/3′-untranslated region (5′/3′-UTR), and the open reading frame (ORF). (**b**) 5′ Regulatory region sequence of the bovine *SIX1* gene. Arrows mark the transcription initiation sites. The translational start site (ATG) is shown in red letters. The transcription factor binding sites are boxed, and primer sequences are underlined with the respective names shown below the line. The CpG island is indicated by a blue box. (**c**) Schematic representation of the relative loci of the E-box motif, MyoD, CEBP, SIX4, CREB, PAX7, MyoD, MEF2, TEAD4 and MEF3 motif binding sites in the *SIX1* promoter. Arrows mark the motifs and transcription factors recognition sites. Dashed lines indicate the GC percentage as represented on the y-axis and the x-axis denotes the bp position in the 5′ untranslated region. Coordinates are given relative to the translational start site (shown as +1).

The bovine *SIX1* genes spans approximately 4.76 kb on chromosome 10 and contains two exons and one intron (Fig. 2a). Based on the bovine *SIX1* cDNA sequence (GenBank No. XM_588692.7), we identified an open reading frame (ORF) of 855 bp, which encoded 284 amino acids (aa) with a calculated molecular weight of 32.18 kDa and an isoelectric point (pI) of 9.24. In addition, the bovine SIX1 protein contains two putative domains: the N-terminal SD domain and the homeodomain. The N-terminal SD domain resides in aa 9 to 118, and the homeodomain resides in aa 125 to 186 (Fig. 2a). The bovine SIX1 aa sequence was highly similar with other mammalian proteins, with the following levels of sequence similarity: goat (99%), pig (99%), dog (99%), human (99%), mouse (98%) and chicken (92%). The phylogenetic tree indicated that of all six species evaluated for this study, bovine SIX1 was most closely related to goat and was least similar to chicken (Fig. 1c). This result indicates that the *SIX1* gene is highly homologous across species and is characterized by two stable putative domains.

### Characterization of the bovine SIX1 gene 5′ regulatory region

To identify regulatory elements in the *SIX1* 5′ regulatory region, we cloned and analysed a 2-kb of 5′ regulatory region using the Matlnspector programme with a cut-off value of over 90%. Several potential TF recognition sites were detected, including the E-box binding factors (E-box), CREB, PAX7, MyoD, MEF2 and TEAD4 (Fig. 2b and c). Additionally, computational analysis indicated that the bovine *SIX1* 5′ regulatory region contained neither a consensus TATA box nor a consensus CCAAT box close to the TSS +1 (Fig. 2c), in complete accord with the investigation of the published SIX1 mRNA sequence (XM_588692.7) by 5′ rapid amplification of cDNA end analysis (RACE) in our previous study^18^.

### Transcriptional regulation of the bovine SIX1 gene

**Figure 3:**
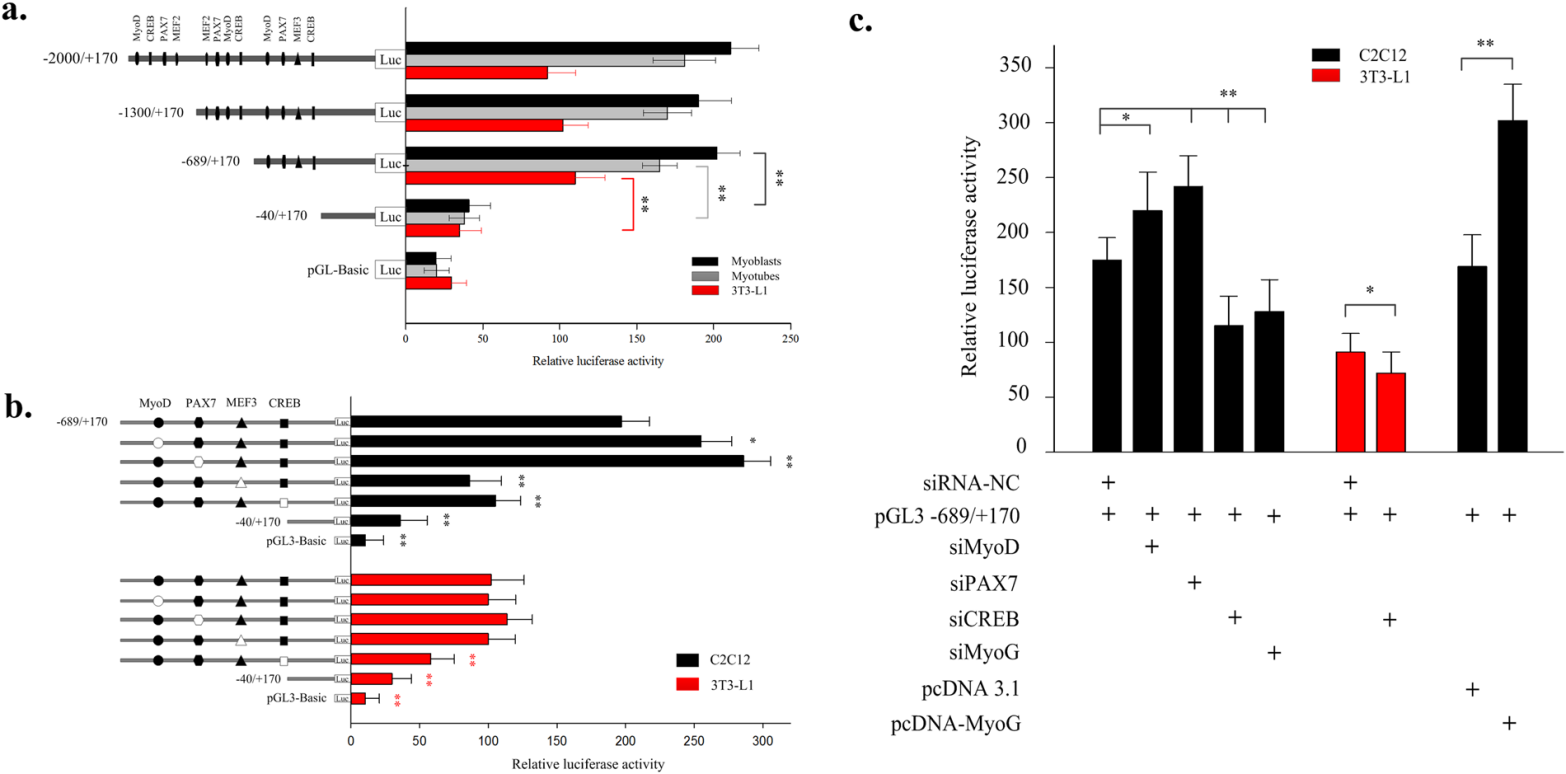
Analysis of MyoD, PAX7, MEF3, CREB and MyoG binding sites by site-directed mutagenesis and siRNA interference of the bovine *SIX1* promoter constructs in two cell lines. (**a**) A series of plasmids containing 5′ unidirectional deletions of the promoter region of the *SIX1* gene (pGL3-P1, pGL3-P2, pGL3-P3, pGL3-P4 and pGL3-basic) fused in-frame to the luciferase gene were transfected into 3T3-L1 and C2C12 cells. After 5 h, we replaced the transfection mixture with DMEM with 2% HS (myotubes). (**b**) Site-directed mutagenesis was carried out in the construct pGL-689/+170. The different constructs were transiently transfected into C2C12 and 3T3-L1 cells. (**c**) MyoD, PAX7, CREB and MyoG knockdown by siRNA co-transfected with pGL-689/+170 in C2C12 or 3T3-L1 cells. The NC siRNA was used as a negative control. MyoG overexpression using the constructed pcDNA3.1 (+) expression plasmid. After 48 h, the cells were harvested for the luciferase assays. The results are expressed as the means ± SD in arbitrary units based on the firefly luciferase activity normalized against the Renilla luciferase activity for triplicate transfections. The error bars denote the standard deviation. Paired Student’s t-test was used to detect significant differences. *P < 0.05 and **P < 0.01. Data are shown as the means ± SD (n = 3).

To determine the minimum sequence required for activity and identify the activity of potential TFs for the bovine SIX1 gene in the 5′ regulatory region, we generated four serial reporter constructs in pGL3-basic containing −2000/+170, −1300/+170, −689/+170 and −40/+170, which represent with progressively larger deletions from the 5′ end of the promoter. The effects of these modifications were evaluated based on transfection of the corresponding luciferase reporter plasmids into undifferentiated and differentiated C2C12 and 3T3-L1 cells. The results of these analyses are shown in Fig. 3a. The luciferase assays revealed 10.8-fold, 9.2-fold and 3.5-fold increased promoter activity of pGL-2000/+170 compared with empty vector in undifferentiated and differentiated C2C12 and 3T3-L1 cells, respectively, indicating a functional promoter in the −2000/+170 region of the *SIX1* gene. When the promoter was deleted to position −40 in pGL-40/+170, the promoter activity decreased by approximately 80.2%, 76.9% and 76.8% in undifferentiated and differentiated C2C12 and 3T3-L1 cells compared with pGL-689/+170 respectively. These results suggest that the core functional promoter of the bovine *SIX1* gene is located within the −689/−40 region relative to TSS-1 and that an undifferentiated cell model may be superior for determining the transcriptional activities of the *SIX1* gene (Fig. 3a). Sequence analysis showed potential binding sites for the TFs MyoD, CREB, PAX7 and MEF2 at −2000/−1300, −1300/−689 and −689/−40. The luciferase activity decreased significantly compared with the other serial reporter constructs only when the promoter sequence was deleted as in the −689/−40 fragment (Fig. 3a). Thus, we hypothesized that the potential TFs MyoD, PAX7 and CREB and the MEF3 motif bind in the −689/−40 region and play major roles in regulating the transcriptional activity of the bovine *SIX1* gene.

### Identification of the MyoD (ctatacagctgggagt)-, PAX7 (atcaatccattactt)-, MEF3 (ggctcacgttgca)- and CREB (gcaaggtcctgacgcgctcac)- binding sites as transcriptional activators or repressors

**Figure 4:**
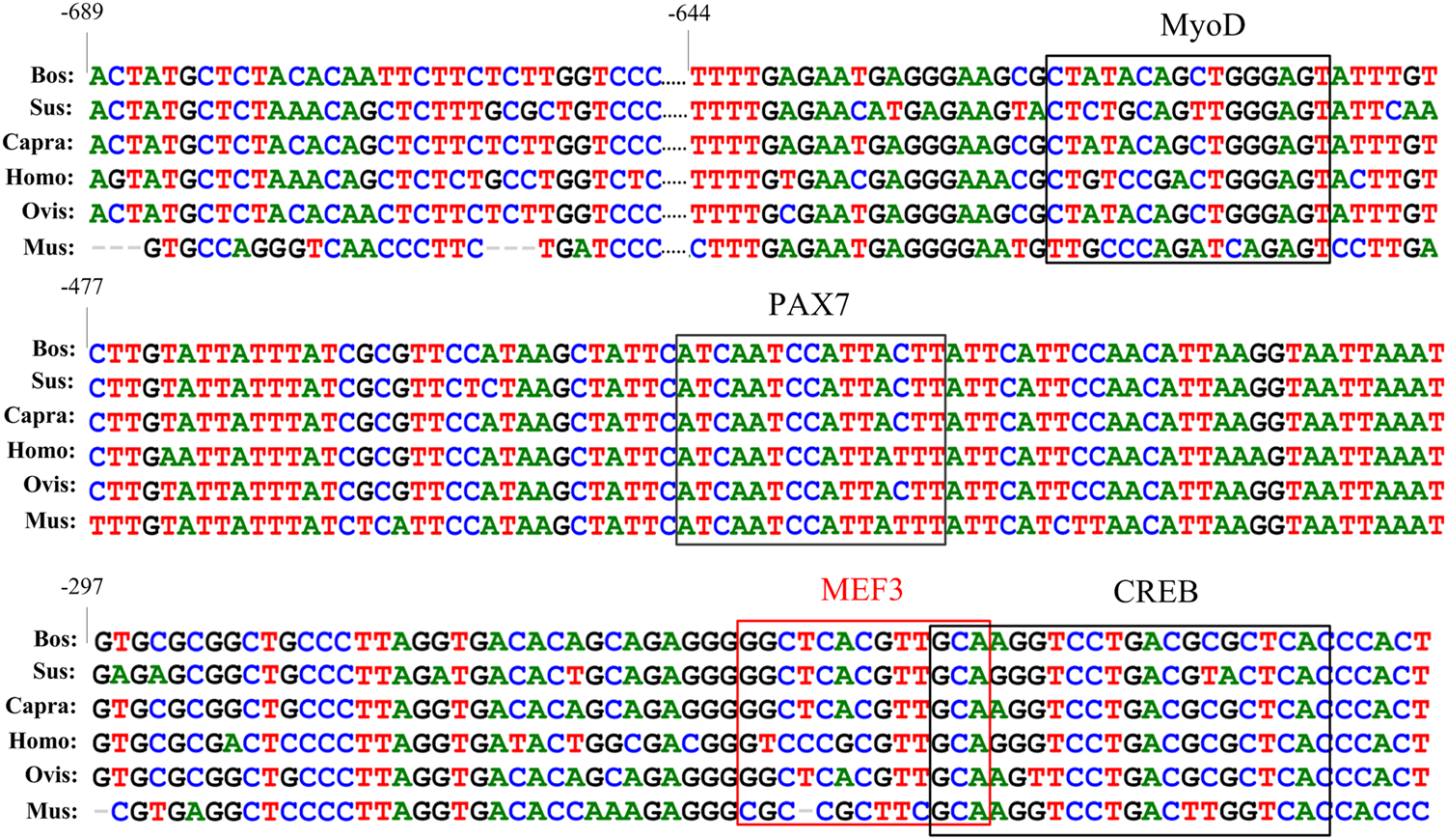
Multi-alignment sequence analysis of the MyoD, PAX7, MEF3 and CREB transcription factor binding sites in the promoter region of the *SIX1* gene in cattle, pigs, goats, humans, sheep, and mice.

To investigate the roles of these sites in the regulation of *SIX1*, we constructed a series of DNA plasmids with 4-bp point mutations in the TF binding sites and transiently transfected them into C2C12 and 3T3-L1 cells. As shown in Fig. 3b, mutation of the MyoD or PAX7 site in the construct pGL-689/+170 resulted in a significant increase in *SIX1* promoter activity of 123% or 145%, respectively, in C2C12 cells. Mutations of the MEF3 or CREB site in the construct pGL-689/+170 led to approximately 47–56% reductions in *SIX1* promoter activity in C2C12 cells. By contrast, mutation of MyoD, PAX7 and MEF3 had no effect on *SIX1* promoter activity in 3T3-L1 cells, whereas mutation of CREB in 3T3-L1 cells led to a decrease in *SIX1* promoter activity of 43% (Fig. 3b). Further validating these potential TFs, co-transfection of siRNAs against MyoD and PAX7 into C2C12 cells dramatically increased *SIX1* transcription levels (125% and 138%, respectively) (Fig. 3c). However, siRNA against CREB led to significant decrease in *SIX1* promoter activity of 34% and 21% in C2C12 and 3T3-L1 cells, respectively. Moreover, MyoG knockdown and overexpression significantly altered the level of *SIX1* promoter activity in C2C12 cells (a 29% decrease and 178% increase, respectively, Fig. 3c). Thus, we hypothesized that MyoG be responsible for this regulatory activity via the MEF3 motif according to previous reports that MyoG interacts with *SIX1* via MEF3 motifs during embryonic development^1^. In addition, multi-alignments among the six species were performed. The results revealed conservation of MyoD, PAX7, MEF3 and CREB elements in this region for among domestic animals such as cattle, goats and pigs, as well as humans (Fig. 4).

### MyoD, PAX7, CREB and MyoG bind to the *SIX1* promoter in *vitro* and *vivo*

**Figure 5:**
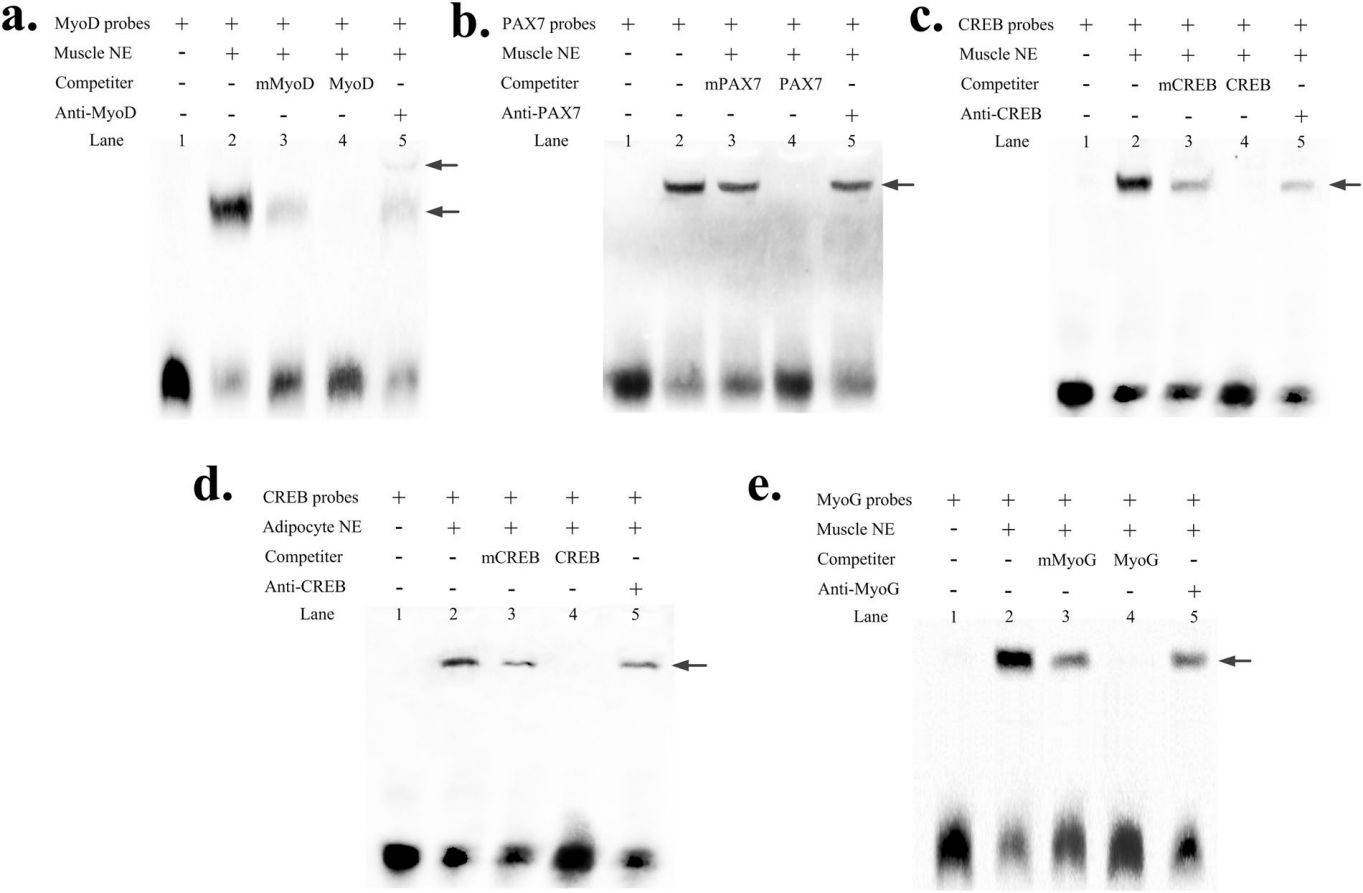
EMSAs showing direct binding of MyoD, PAX7, CREB and MyoG to the *SIX1* promoter in *vitro*. Nuclear protein extracts were incubated with 5′-biotin labelled probe containing the MyoD, PAX7, CREB or MyoG binding site in the presence or absence of competitor (lane 2), 50× mutation probe (lane 3) and 50× unlabelled probes (lane 4). The super-shift assay was conducted using 10 μg of anti-MyoD, anti-PAX7, anti-CREB or anti-MyoG antibodies (lane 5). The arrows mark the main complexes. Muscle NE, QCMC nuclear protein extracts (**a**,**b**,**c** and **d**). Adipocytes NE, adipocyte nuclear protein extracts (**e**).

**Figure 6:**
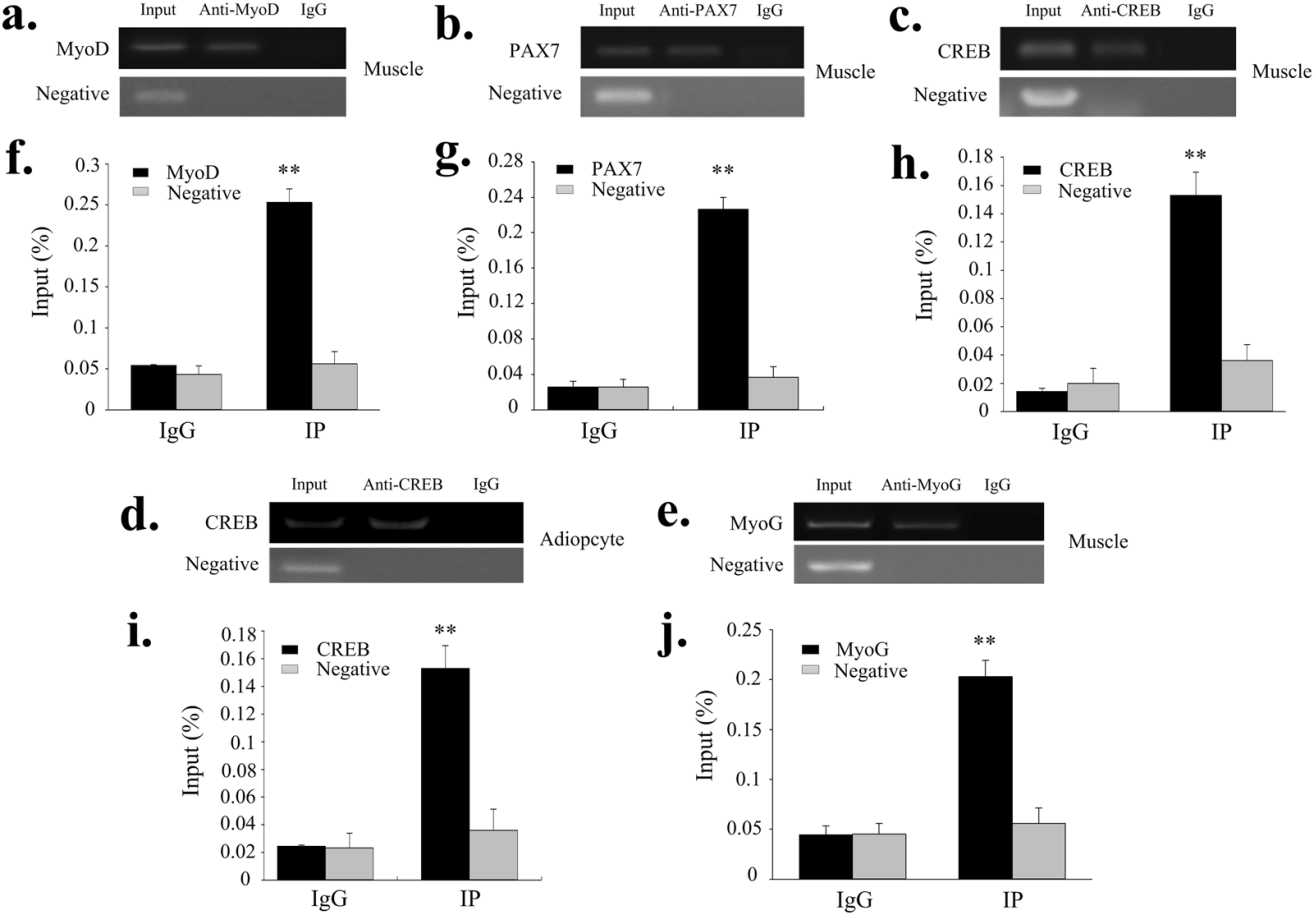
ChIP assay of MyoD, PAX7, CREB and MyoG binding to the *SIX1* promoter in *vivo*. ChIP-PCR products were amplified with the indicated primers using input and immunoprecipitated products for MyoD (**a**), PAX7 (**b**), CREB (**c**,**d**) and MyoG (**e**) from muscle and adipocyte. ChIP-qPCR assays detected the enrichment of DNA fragments in samples immunoprecipitated with MyoD (f), PAX7 (**g**), CREB (**h**,**i)** and MyoG (**j**) antibodies. Total chromatin from muscle (**a**,**b**,**c**,**e**,**f** and **j**) and adipocytes (d and i) was used as the input. Normal rabbit IgG and an intragenic DNA fragment of *SIX1* exon 2 were used as negative controls. **P < 0.01. Error bars represent the SD (n = 3).

We used EMSAs and ChIP assays to determine if MyoD, PAX7, CREB and MyoG bind to the *SIX1* promoter both in *vitro* and *vivo*. As shown in Fig. 5, nuclear protein from Qinchuan cattle myoblast cells (QCMCs) bound to the 5′-biotin labelled MyoD probes and formed one main complex (lane 2, Fig. 5a). Competition assays verified that the mutant probe had little effect on this complex (lane 3, Fig. 5a). However, the specific of the MyoD/DNA interaction was prevented by competition from excess non-labelled DNA (lane 4, Fig. 5a). The last lane shows that the complex was super-shifted upon incubation with a MyoD-antibody (lane 5, Fig. 5a). PAX7 and MyoG yielded results similar to those of MyoD in the QCMC nuclear extracts (Fig. 5b and c). Similar results were also obtained for the CREB site in QCMCs and adipocyte nuclear extracts (Fig. 5d and e). Although the PAX7, MyoG and CREB EMSAs did not reveal a super-shifted product at the binding sites, the upshifted bands were diminished when the antibodies were added and incubated. The super-shift may be formed a high-molecular-weight polymer and be stuck in the top of the well, causing reduction gel mobility shift (lane 5, Fig. 5b,c,d and e). The ChIP results revealed that MyoD, PAX7, CREB and MyoG interacted with the binding sites (Fig. 6a,b,c,d and e), and the relative enrichment levels were ~6.3-, ~8.7-, ~4.7- to 8.9- and 4.6-fold over the IgG control respectively (Fig. 6f,g,h,i and j), based on three independent experiments.

## Discussion

The *SIX1* gene plays an important role in embryonic myogenesis and has very high expression levels and specific expression patterns in the skeletal muscle of pigs, humans and ducks^19–21^. In the present study, bovine *SIX1* was found to be highly expressed in the *longissimus thoracis* muscle, thereby indicating that the *SIX1* gene might play a functional role in mediating the development of bovine skeletal muscle. The bovine *SIX1* aa sequence shares a 99% similarity with sequences in the goat, pig, dog and human, and bovine *SIX1* contains two putative domains: the N-terminal SD domain and the homeodomain. These results indicate that the function of *SIX1* is primarily coordinated by specific DNA-binding factors and cooperative interactions with co-factors^8^. Additionally, these results show that the *SIX1* gene is highly conserved and has similar functions in ruminants.

To further elucidate the regulation of the bovine *SIX1* gene at the transcriptional level, we cloned and analysed its 5′ regulatory region. The results revealed that there was no consensus TATA box or CCAAT box; however, there was a CpG island containing the promoter region from −235 to +658 relative to the TSS. Consistent with these observations TATA boxes are enriched in a minority of mammalian gene promoters^22,23^, and only 10–20% of mammalian promoters contain a functional TATA box^23,24^. DNA methylation is involved in stable gene silencing (for example, on the inactive X chromosome), either through interference with TF binding or through the recruitment of repressors that specifically bind sites containing methylated CG^25,26^. A previously a study has shown that the porcine *SIX1* gene is highly associated with DNA methylation during myoblast differentiation^27^. Thus, we hypothesized that the GC-rich region of the bovine *SIX1* gene promoter is subject to epigenetic regulation.

An analysis of the region comprising positions −2000 to + 170 of the bovine *SIX1* gene using online prediction software identified potential TF binding sites for MyoD, CREB and PAX7 at −2000/−1300, −1300/−689 and −689/−40. However, the luciferase activity decreased significantly compared with the other serial reporter constructs only when the promoter sequence was deleted in the −689/−40 fragment. Therefore, we hypothesized that the potential TFs MyoD, PAX7 and CREB and MEF3 motif at −689/−40 to play major roles in regulating the transcriptional activity of the bovine *SIX1* gene. MyoD belongs to the MRF family and plays key roles in muscle plasticity and regeneration. MyoD expression is strongly induced early after injury as satellite cells become activated in part through the action of the TF *SIX1* ^1,28^. Additionally, MyoD co-expressed with PAX7 regulates satellite cell physiology by inducing self-renewal^29^ and maintains a population of functional satellite cells in the undifferentiated state^30^. In the absence of MyoD and PAX7, satellite cells die and thus fail to repopulate, leading to severe skeletal muscle defects^31^. Furthermore, MyoD and PAX7 expression levels are increased by activation of the Notch signalling pathway, and increased MyoD and PAX7 expression levels promote self-renewal and proliferation while inhibiting differentiation^32,33^. However, *SIX1* overproduction represses proliferation and promotes the differentiation of satellite cells. There appears to be some distinction between MyoD and PAX7 regarding the function of *SIX1* in the postnatal stage of myogenesis development^28^. In the present study, MyoD and PAX7 mutation and knock down increased the basal promoter activity. The EMSA and ChIP results showed that MyoD and PAX7 were capable of binding to *SIX1* with high affinity in C2C12 cells, thereby suggesting that MyoD and PAX7 may have compensatory mechanisms in the function of the *SIX1* gene and may contribute to determining the fate of bovine skeletal muscle cells.

CREB plays key roles in the differentiation of embryonic skeletal muscle progenitors and in the survival of adult skeletal muscle^34^. Moreover, signals from damaged skeletal muscle tissues induce CREB phosphorylation and target gene expression in primary mouse myoblasts. Activated CREB localizes to both myogenic precursor cells and newly regenerating myofibres within regenerating areas^35^. Additionally, a previous study showed that CREB can interact directly with MyoD, inducing transactivation attenuated by interference with its dimerization^36^. CREB is constitutively expressed in 3T3-L1 fibroblasts and regulates the adipocyte-specific genes phosphoenol pyruvate carboxykinase (PEPCK), fatty acid binding protein (FABP (aP2/422)), fatty acid synthetase (FAS) andcyclooxygenase (COX)−2, which induce adipogenesis as “adipocyte-specific” markers during the early phase of adipogenesis^37,38^. In the present study, we observed that CREB mutation and knock down reduced the transcriptional activity of the *SIX1* gene in C2C12 and 3T3-L1 cells, respectively, while the EMSA and ChIP results showed that CREB could bind to this sequence. These results indicate that CREB plays an important role in regulating the expression of the bovine *SIX1* gene in skeletal muscle and adipocyte cells.

**Figure 7:**
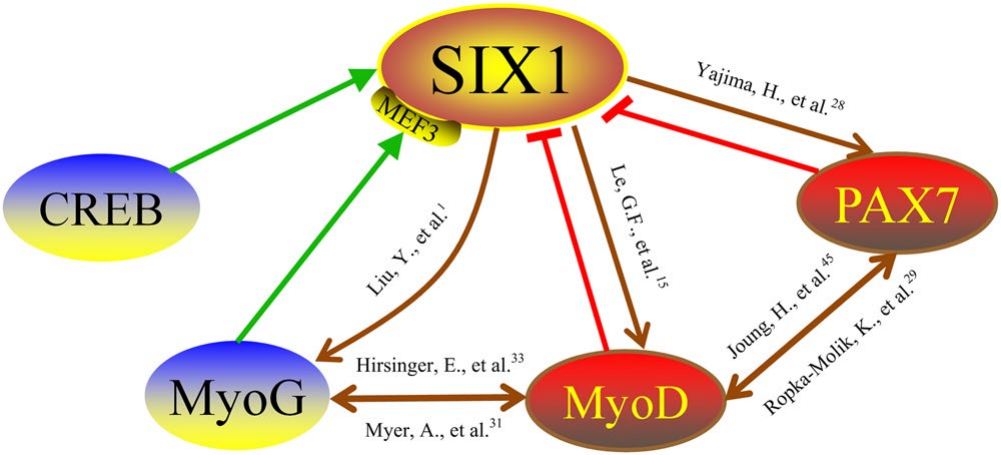
A proposed schematic summary of the interactions of the *SIX1* gene. MyoD, PAX7, CREB and MyoG coordinated with the *SIX1* and form a feed-forward regulatory network. The brown arrows in this diagram represent the interactions of the TFs based on previous reports. The green and red arrows indicate the interactions of the TFs with the *SIX1* gene proposed in the present study.

Another important motif is MEF3, which the most abundant of the MRFs including MyoD and MyoG. MEF3 initiates the expression of muscle-specific proteins by inducing target genes^39,40^. Strikingly, the transcriptional activity of the *SIX1* gene decreased upon MEF3 motif mutation. However, MyoD binds to the −689/−40 promoter fragment of *SIX1* and is a negative regulator. Therefore, we hypothesized that MyoG may have been responsible for this regulatory activity. MyoG is a muscle-specific TF and is up-regulated during myoblast differentiation into multinucleated myotubes^41^. A recent study reported that MyoG interacts with *SIX1* via the E-box and MEF3 motifs during embryonic development and plays a role that is parallel to that played by the SIX family during terminal differentiation of myoblasts^1,40^. Our results strongly support this hypothesis since MEF3 mutation or MyoG knock down led to a significant reduction in the basal activity of the −689/+170 promoter region. However, MyoG overexpression resulted in a boost in promoter activity. Although there is no evidence of potential MyoG TF binding sites in the −689/+170 promoter region of the bovine *SIX1* gene, the EMSA and ChIP results showed that MyoG could bind with high affinity to the MEF3 motif. Taking these results together, we conclude that MyoG plays an important role in regulating *SIX1* indirectly via the MEF3 motif. In addition, the MRFs and the *SIX1* TF form a feed-forward regulatory network that is responsible for inducing the transcription of many muscle function genes, as shown in the proposed model in Fig. 7. This proposed model could be used to further study the interactions of TFs with the bovine *SIX1* gene.

In summary, we cloned the promoter and CDS sequences of the bovine *SIX1* gene and identified its transcription initiation sites. The aa sequence of bovine SIX1 shares high similarity with its homologues in goat and sheep. The *SIX1* gene is highly expressed in the *longissimus thoracis* muscle and is regulated by multiple TFs, including MyoD, PAX7, CREB and MyoG. Epigenetic modifications in the *SIX1* promoter and their effects on the transcription of the gene remain to be investigated. Our results provide a foundation for a better understanding of the transcriptional regulation and biological function of the bovine *SIX1* gene.

## Materials and Methods

### Ethics Statement

All animal procedures were performed according to guidelines laid down by the China Council on Animal Care, and the protocols were approved by the Experimental Animal Manage Committee (EAMC) of Northwest A&F University.

### Quantitative PCR analysis of gene expression patterns

Ten tissues (heart, liver, spleen, lung, kidney, abomasum, small intestine, abdominal fat, *longissimus thoracis* muscle and testicular tissue) were obtained from three Qinchuan foetal bovine. *β-actin* was used as the endogenous reference. Total RNA and cDNA were obtained using an RNA kit and PrimeScript™ RT Reagent kit (Perfect Real Time) (TaKaRa, Dalian, China), respectively. qPCR reaction mixtures (20 μL) contained SYBR Green Real-time PCR Master Mix (TaKaRa) and gene-specific primers (Table 1). The PCR conditions consisted of an initial incubation of 5 min at 95 °C, followed by 34 cycles of 30 s at 95 °C, 30 s at 60 °C and 30 s at 72 °C. Reactions were run in triplicate using a 7500 System SDS V 1.4.0 thermocycler (Applied Biosystems, USA). The relative expression levels of the target mRNAs were calculated using the 2^−ΔΔCt^ method^42^.

**Table 1:** Primers utilized in this study.

Reaction	Name	Primer Sequence (5′ to 3′)	Tm (°C)	Product Length (bp)	Amplified Region	Accession numbers
RT-PCR	*β-actin*	F: CACCAACTGGGACGACAT	60.0	202	320–521	AY141970.1
		R: ATACAGGGACAGCACAGC				
	*SIX 1*	F: GCCAAGGAAAGGGAGAACA	60.0	127	866–992	XM_588692.7
		R: GACTCTGGGGAGGTGAGAACT				
CDS	*SIX1* CDSF/R	F: ATGTCGATGCTGCCATCGTTC	62.0	855	317–1171	XM_588692.7
		R: TTAGGACCCCAAGTCCACCA				
Promoter cloning	*SIX1*-PF/PR	F: ATGAGCACTAAAATAAGACCATGAGGG	65.5	2171	−2000/+170	NC_007308.6
		R: CTTGAAGACTGGAGGCCGAGTA				
	*SIX1*-P1	F: *CGGGGTACC*ATGAGCACTAAAATAAGACC	64.0	2171	−2000/+170	
	*SIX1*-P2	F: *CGGGGTACC*ATCTCCTGCCAGTCTCAGCTAC	61.5	1471	−1300/+170	
	*SIX1*-P3	F: *CGGGGTACC*ACTATGCTCTACACAATTCTTCTC	63.5	860	−689/+170	
	*SIX1*-P4	F: *CGGGGTACC*CTGGCCAGCCCCGGATAGGCG	65.0	211	−40/+170	
	*SIX1*-R	R: *GGAAGATCT*CTTGAAGACTGGAGGCCGAGTA				
Site-mut and EMSA	MyoD forward	GGAAGCGCTATACA*GCTG*GGAGTATTTGTGCGCT			−630/−596	
	MyoD reverse	AGCGCACAAATACTCC*CAGC*TGTATAGCGCTTCC				
	mMyoD forward	GGAAGCGCTATACA*AAAA*GGAGTATTTGTGCGCT			−630/−596	
	mMyoD reverse	AGCGCACAAATACTCC*TTTT*TGTATAGCGCTTCC				
	PAX7 forward	CATAAGCTATTCATC*AATC*CATTACTTATTCATT			−455/−431	
	PAX7 reverse	AATGAATAAGTAATG*GATT*GATGAATAGCTTATG				
	mPAX7 forward	CATAAGCTATTCATC*TTTT*CATTACTTATTCATT			−455/−431	
	mPAX7 reverse	AATGAATAAGTAATG*AAAA*GATGAATAGCTTATG				
	CREB forward	CACGTTGCAAGGTCC*TGAC*GCGCTCACCCACTCC			−259/−225	
	CREB reverse	GGAGTGGGTGAGCGC*GTCA*GGACCTTGCAACGTG				
	mCREB forward	CACGTTGCAAGGTCC*TTTT*GCGCTCACCCACTCC			−259/−225	
	mCREB reverse	GGAGTGGGTGAGCGC*AAAA*GGACCTTGCAACGTG				
	MEF3 forward	CACAGCAGAGGGGGC*TCAC*GTTGCAAGGTCCTGA			−274/−240	
	MEF3 reverse	TCAGGACCTTGCAAC*GTGA*GCCCCCTCTGCTGTG				
	mMEF3 forward	CACAGCAGAGGGGGC*AAAA*GTTGCAAGGTCCTGA			−274/−240	
	mMEF3 reverse	TCAGGACCTTGCAAC*TTTT*GCCCCCTCTGCTGTG				
ChIP	ChIP-MyoD	F: GCTCAGCAACTATGCTCTACA	60	112	−697/−586	
		R: CGCCCTCCAAAGCGCACAAAT				
	ChIP-PAX7	F: GAGGACTGGTCTACGCTTGTAT	60	124	−491/−367	
		R: CTTTGCAGACTTGCCATTTCC				
	ChIP-CREB	F: GAGCCATTGATTTGTGCAAAGATG	60	165	−319/−154	
		R: GCGAGTGGTGAGAAGCGAAGTG				
	ChIP-MyoG	F: GAGCCATTGATTTGTGCAAAGATG	60	165	−319/−154	
		R: GCGAGTGGTGAGAAGCGAAGTG				
	ChIP-control	F: GTTTTGTTTACCACTAGCTTTTC	60	134	*SIX1* exon 2	
		R: ATCCTTGTAGGAGTTCCCTTT				
siRNA	siMyoD	CCAAUGCGAUUUAUCAGGUGCUUUGTT				
	siPAX7	GGUAACAUCCCAGCUUUACTT				
	siCREB	AAUACAGCUGGCUAACAAUGGTT				
	siMyoG	AACUACCUUCCUGUCCACCTT				
	siRNA-NC	UUCUCCGAACGUGUCACGUTT				
Overexpression	*MyoG*-CDSF/R	F: *CCCAAGCTT*ATGGAGCTGTATGAGACCTCTC	62.5	675		NM_001111325.1
		R: *CTAGTCTAGA*TCAGTTTGGTATGGTTTCATCTG				

The italicized letters of the forward (F) and reverse (R) primers for promoter cloning and overexpression items indicate the enzyme cutting sites of promoter cloning (*Kpn*I and *Bgl*II) and overexpression items (*Hind*III and *Xbal*), respectively. The underlined bases are core putative transcription factor-binding sites.

### Western blotting

Tissues protein was extracted using T-PER Tissue Protein Extraction Reagent (Pierce, Thermo Fisher Scientific, USA). The total protein samples were quantified using the Pierce BCA Protein Assay Kit (Thermo Scientific) and 50 μg of total protein was separated by electrophoresis on a 10% SDS-polyacrylamide gel, followed by transfer to nitrocellulose. After blocking in defatted milk powder, the membranes were incubated with the SIX1 antibody (sc-514441, Santa Cruz, USA) and β-actin antibody (ab 8226, Abcam, USA). The blots were washed and subsequently treated with a peroxidase labelled secondary antibody. The signals were detected by exposure of X-ray films to chemical luminescence using the ChemiDoc™ XRS+ System (Bio-Rad, Hercules, CA, USA).

### Molecular cloning and sequence analysis

The complete *SIX1* CDS was cloned from *longissimus dorsi* muscle cDNA using specific primers (*SIX1*-CDSF/R, Table 1). The gene-specific primers (*SIX1*-PF/PR, Table 1) were designed to amplify a 2-kb promoter region, including the translational start site, of the bovine *SIX1* gene (NCBI accession AC_000167.1 from 73068130 to 73074897). PCR amplifications were performed using genomic DNA from Qinchuan cattle blood as a template, using KOD DNA Polymerase (Toyobo, Osaka, Japan) to amplify the 5′-regulatory region sequence. The potential TF binding sites were analysed using the Genomatix suite (http://www.genomatix.de/). CpG islands were predicted using MethPrimer (http://www.urogene.org/methprimer/). Structural and phylogenetic tree analyses of *SIX1* were performed using the SMART database (http://smart.embl-heidelberg.de/) and MEGA 5.1 (http://www.megasoftware.net), respectively.

### Promoter cloning and generation of luciferase reporter constructs

The fragment primers *SIX1*-P1/R (−2000/+170), *SIX1*-P2/R (−1300/+170), *SIX1*-P3/R (−689/+170) and *SIX1*-P4/R (−40/+170) were designed to contain unidirectional deletions of the bovine *SIX1* promoter. The PCR complexes were generated using specific primers that included the sequences of the *Kpn*I and *Bgl*II restriction sites and the *SIX1*-PF/PR products as a template. PCR fragments were then cloned into the pMD19-T (simple) vector (Takara) and ligated into the luciferase reporter construct pGL3-basic vector digested with the same restriction enzymes *Kpn*I and *Bgl*II (Takara). These plasmids were named pGL3-P1, pGL3-P2, pGL3-P3 and pGL3-P4.

### Cell culture and transfection

C2C12 and 3T3-L1 cells were maintained in Dulbecco’s Modified Eagle Medium (DMEM) supplemented with 10% newborn calf serum (NBCS; Invitrogen, USA) and antibiotics (100 IU/mL penicillin and 100 µg/mL streptomycin) at 37 °C and 5% CO_2_ in an atmospheric incubator. Cells were grown overnight in 24-well plates with growth medium without antibiotics until reaching 80–90% confluence at a density of 1.2 × 10^5^ cells. In each well, the transfection reagent was mixed with 800 ng of the expression construct (pGL3-P1, pGL3-P2, pGL3-P3 or pGL3-P4), 10 ng of pRL-TK normalizing vector, and 2 μL of X-tremeGENE HP DNA transfection reagent (Roche, USA); the samples were then incubated with 100 μL of opti-DMEM (GIBCO; Invitrogen). The pGL3-basic vector served as a negative control. At 6 h after transfection, we replaced the media with DMEM with 2% horse serum (HS) (GIBCO, Invitrogen) and incubated for 42 h to induce differentiation of the C2C12 myoblasts into myotubes. Cell lysates were collected 48 h post-transfection and used for the measurement of the relative transcriptional activity of each fragment with the Dual-Luciferase Reporter Assay System (Promega, USA), according to the manufacturer’s instructions. The relative luciferase activities were determined using a NanoQuant Plate™ (TECAN, infinite M200PRO). Experiments were conducted in parallel and in triplicate.

### Site-directed mutagenesis

The potential TF-binding sites for MyoD, PAX7, MEF3 and CREB motif were mutated with the corresponding primers (Table 1) using the Quick Change Site-Directed Mutagenesis Kit (Stratagene, La Jolla, CA, USA). The PCR was carried out using the following conditions: 98 °C for 2 min; 34 cycles of 97 °C for 15 s, 60 °C for 10 s, and 68 °C for 5 min; and finally, 68 °C for 5 min. The reaction product was treated with *Dpn*I and then amplified with XL10-Gold competent cells (Stratagene) and sequenced.

### MyoD, PAX7, CREB and MyoG knockdown and MyoG overexpression

siRNAs against MyoD, PAX7, CREB and MyoG were designed as described previously^43–46^ and synthesized with the control siRNA (GenePharma Co., Ltd., Shanghai, China). The NC siRNA served as a negative control. The siRNA sequences are presented in Table 1. C2C12 and 3T3-L1 cells cultured in 24-well plates were transiently co-transfected with 50 nM each siRNA and the corresponding pGL3–689/+170. The pcDNA3.1-MyoG expression plasmid was generated by reverse PCR to obtained the bovine myogenin CDS (NCBI: NM_001111325.1) using specific primers (Table 1) that included *Hind*III and *Xbal* restriction sites to allow ligation into the pcDNA3.1 vector digested with these restriction enzymes. For MyoG overexpression, C2C12 cells cultured in 24-well plates were transiently co-transfected with 400 ng each of pcDNA3.1-MyoG and of the corresponding pGL3 −689/+170. The pcDNA3.1 plasmid served as a negative control.

### Electrophoretic mobility shift assays (EMSAs)

Qinchuan cattle myoblast cells (QCMCs) and adipocytes were isolated from Qinchuan foetal bovine samples as described previously^47^. To obtain nuclear extracts, QCMCs and adipocytes were treated using the Nuclear Extract kit (Active Motif Corp., Carlsbad, CA, USA) according to the manufacturer’s protocol. A LightShift Chemiluminescent EMSA Kit (Thermo Fisher Corp., Waltham, MA, USA) was used for the EMSAs according to the manufacturer’s protocol with modifications. Briefly, 200 fmol of 5′-biotin labelled probe (listed in Table 1) was incubated with a reaction mixture; containing 2 μL of 10× binding buffer, 1 μL of poly (dI.dC), 1 μL of 50% glycerol and 10 μg of nuclear protein extract in a 20 μL total volume. For the competition assay, unlabelled or mutated DNA probes were added to the reaction mixture for 15 min before adding the labelled probes. For the super-shift assay, 10 μg each of MyoD (sc-31940), PAX7 (sc-365843), CREB (sc-377154) or Myogenin (sc-52903) (Santa Cruz, USA) antibodies were added to the reaction mixture. Then, the reaction mixture was incubated on ice for 30 min, after which the labelled probes were added. Finally, the DNA-protein complexes were separated on a 6% non-denaturing polyacrylamide gel by polyacrylamide electrophoresis (PAGE) using 0.5 × TBE buffer for 1 h. Images were captured using the molecular imager ChemiDoc™ XRS+ system (Bio-Rad).

### Chromatin immunoprecipitation (ChIP) assay

The ChIP assays were performed using the SimpleChIP® Enzymatic Chromatin IP kit (CST, Massachusetts, USA) according to the manufacturer’s protocol. QCMCs and adipocytes from Qinchuan foetal bovine samples (n = 3) were used. The protein-DNA complexes were cross-linked with 37% formaldehyde and neutralized with glycine. After digestion of the DNA with micrococcal nuclease into fragments of approximately 150–900 bp in length, the fragmented chromatin samples were suspended in ChIP dilution buffer as an input. The cross-linked chromatin samples were immunoprecipitated with 4 μg of MyoD, PAX7, CREB or MyoG antibodies and with normal rabbit IgG overnight at 4 °C. The immunoprecipitated products were isolated with protein G agarose beads, and the bound chromatin was then collected by salt washing. The eluted ChIP Elution Buffer was then digested with proteinase K and purified for PCR analysis. All ChIP primers used in the standard PCR and quantitative real-time PCR experiment are listed in Table 1. The ChIP-PCR reaction mixtures had a total volume of 20 μL containing 10 μL of PCR Mix (TaKaRa), 0.4 μM each primers and 50 ng of template DNA and were performed under the following conditions: initial incubation of 5 min at 95 °C, followed by 34 cycles of 30 s at 95 °C, 30 s at 60 °C and 30 s at 72 °C. The input, immunoprecipitated and normal rabbit IgG products were added to each tube as a template. Amplification was verified by electrophoresis of the products in a 2% (w/v) agarose gel. ChIP-qPCR reaction mixtures (20 μL) contained SYBR Green Real-time PCR Master Mix (TaKaRa) and gene-specific primers. The amplifications were performed in a 7500 System SDS V 1.4.0 thermocycler (Applied Biosystems) with the following conditions: initial denaturation at 95 °C for 5 min followed by 35 cycles of denaturation at 94 °C for 30 s, annealing at 60 °C for 30 s, and extension at 72 °C for 30 s. The percent input was calculated as follows: % Input = 2^[−ΔCt(Ct[ChIP]−(Ct[Input]−Log2(Input Dilution Factor)))]^ ^48^. We used normal rabbit IgG and intragenic DNA fragment of *SIX1* exon 2 as negative controls.

## Electronic supplementary material

Western blots showing the different of bovine Six1 expression in tissues and organs and control.
