# Supplementary material for: Characterization of the promoter region of the bovine SIX1 gene: Roles of MyoD, PAX7, CREB and MyoG
Source: Sci Rep. 2017 Oct 3;7:12599. doi: 10.1038/s41598-017-12787-5 (PMC5626756; doi:10.1038/s41598-017-12787-5)
Supplement: Supplementary file 1 — Western blots showing the different of bovine Six1 expression in tissues and organs and control. [file 41598_2017_12787_MOESM1_ESM.pdf]

Characterization of the promoter region of the bovine *SIX1* gene: Roles of MyoD, PAX7, CREB and MyoG

Da-wei Wei[1], Xue-yao Ma[1], Song-Zhang[1], Jie-yun Hong[1], Lin-sheng Gui[1,3], Chu-gang Mei[1,3], Hong-fang Guo[1], Li-Wang[1], Yue-Ning[1] & Lin-sen Zan[1,2,4*]

[1]College of Animal Science and Technology, Northwest A&F University, Yangling 712100 Shaanxi, People's Republic of China. [2]National Beef Cattle Improvement Center, Northwest A&F University, Yangling 712100 Shaanxi, People's Republic of China. [3]Modern Cattle Biotechnology and Application of National-Local Engineering Research Center, Yangling 712100 Shaanxi, People's Republic of China. [4]Shaanxi Beef Cattle Engineering Research Center, Yangling 712100 Shaanxi, People's Republic of China.

Correspondence and requests for materials should be addressed to L.-S.Z. (email: zanlinsen@163.com). Tel.: +86-29-8709-1923; Fax: +86-29-8709-2164.

SUPPLEMENTARY INFORMATION includes:

Supplementary Figures S1

Supplementary Flies of the Ethic Committee

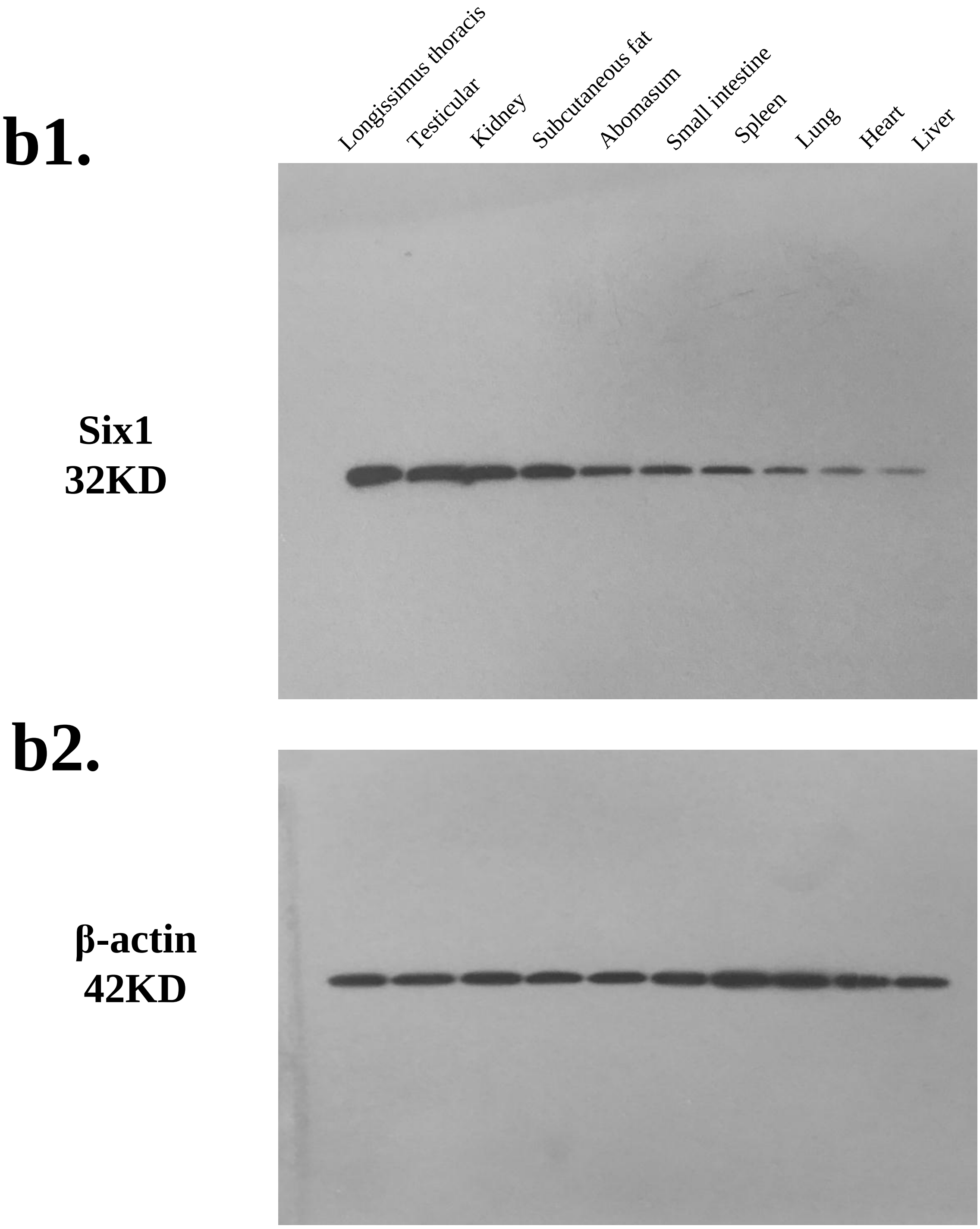

Supplementary Figure S1: Western blots showing the different of bovine Six1 expression in tissues and organs and control.
